# Randomized, Controlled Trial Evaluating a Baby Wash Product on Skin Barrier Function in Healthy, Term Neonates

**DOI:** 10.1111/1552-6909.12015

**Published:** 2013-02-19

**Authors:** Tina Lavender, Carol Bedwell, Stephen A Roberts, Anna Hart, Mark A Turner, Lesley-Anne Carter, Michael J Cork

**Affiliations:** School of Nursing, Midwifery and Social Work, The University of ManchesterManchester, UK; Midwifery and Social Work, The University of ManchesterManchester, UK; The University of ManchesterManchester, UK; The University of LancasterLancaster, UK; University of Liverpool/Liverpool Women's HospitalLiverpool, UK; The University of ManchesterManchester, UK; The University of SheffieldSheffield, UK

**Keywords:** randomized, term neonates, wash product, neonatal skin care, transepidermal water loss, noninferiority trial

## Abstract

**Objectives:**

To examine the hypothesis that the use of a wash product formulated for newborn (<1 month of age) bathing is not inferior (no worse) to bathing with water only.

**Design:**

Assessor-blinded, randomized, controlled, noninferiority trial.

**Setting:**

A teaching hospital in the Northwest of England and in participants’ homes.

**Participants:**

Three-hundred-and-seven healthy, term infants recruited within 48 hours of birth.

**Method:**

We compared bathing with a wash product (*n* = 159) to bathing with water alone (*n* = 148). The primary outcome was transepidermal water loss (TEWL) at 14 days postbirth; the predefined difference deemed to be unimportant was 1.2. Secondary outcomes comprised changes in stratum corneum hydration, skin surface pH, clinical observations of the skin, and maternal views.

**Results:**

Complete TEWL data were obtained for 242 (78.8%) infants. Wash was noninferior to water alone in terms of TEWL (intention-to-treat analysis: 95% confidence interval [CI] for difference [wash–water, adjusted for family history of eczema, neonate state, and baseline] −1.24, 1.07; per protocol analysis: 95% CI −1.42, 1.09). No significant differences were found in secondary outcomes.

**Conclusion:**

We were unable to detect any differences between the newborn wash product and water. These findings provide reassurance to parents who choose to use the test newborn wash product or other technically equivalent cleansers and provide the evidence for health care professionals to support parental choice.

Infant skin has several important functions, including prevention of infection, maintenance of a stable water content level, and reduction of the penetration of allergens and irritants (Holbrook, 2000). These functions depend on the maintenance of an effective skin barrier with an optimum pH (Cork et al., 2008). A neonate's skin remains immature for some time following birth; it has been demonstrated that the stratum corneum continues to develop until at least age 12 months (Nikolovski, Stamatas, Kollias, & Wiegand, 2008). The normal skin pH at the surface of the stratum corneum after the first year of life is around 5.5. This low pH is important for maintaining low protease activity and enhancing the synthesis of the lipid lamellae, which are central to the maintenance of a normal skin barrier (Danby & Cork, 2011).

The most common skin diseases during this first year of life are napkin/diaper dermatitis, skin infections, and atopic dermatitis (AD) (Atherton & Mills, 2004). Atopic dermatitis occurs as a result of gene–environment interactions leading to skin barrier breakdown (Cork et al., 2009). Soap and harsh surfactants play an important role in facilitating skin barrier deterioration and triggering AD onset (Danby & Cork, 2011). The optimal wash product for a neonate should have a pH around 5.5 and some buffering capacity to maintain skin pH around this level. This was the pH of the wash product used in this trial. At the other end of the spectrum, a soap bar can raise the pH of the skin above 8.0. This leads to enhanced protease activity and inhibits the synthesis of the lipid lamellae, resulting in breakdown of the skin barrier (Cork et al.; Danby & Cork). When oils are reacted with a solution of sodium hydroxide, they break down to form glycerol and the sodium salts of their fatty acids. These salts are used as soap, which are an example of an anionic surfactant. Surfactants have varying effects on skin barrier integrity (Goffin, Paye, & Piérard, 1995). Surfactants with a negative charge (e.g., sodium dodecyl [lauryl] sulphate) have greater skin irritation potential compared with glycosylated surfactants (Ananthapadmanabhan, Moore, Subramanyan, Misra, & Meyer, 2004), the latter of which were used in the study wash product.

Atopic dermatitis is a significant health care burden and impairs the quality of life of infants (Lewis-Jones, Finlay, & Dykes, 2001) and their parents (Lawson, Lewis-Jones, Finlay, Reid, & Owens, 1998). Such problems highlight the need for suitable skin care regimens. As a result, water alone has been suggested as the least harmful method for newborn cleansing in many countries (National Institute for Health and Clinical Excellence [NICE], 2006). However, the buffering capacity of water has been questioned, as it may increase the skin surface pH from 5.5 to 7.5 (Tsai & Maibach, 1999). A pH of 7.5 is likely to increase skin protease activity and inhibit the synthesis of the lipid lamellae, leading to a breakdown of the skin barrier (Tsai & Maibach). Water alone has been identified as an ineffective cleanser, as it fails to remove fat-soluble substances such as feces and sebum (Gelmetti, 2001). This is an issue highlighted as particularly important by mothers (Lavender et al., 2009).

Although guidelines exist for the treatment of atopic eczema (NICE, 2007), there are no guidelines for primary prevention of atopic eczema. Within the United Kingdom, national postnatal care guidelines recommend bathing with water alone in the early postnatal period (NICE, 2006). However, neonatal skin care guidelines in the United States (Lund et al., 2007) recommend the use of warm tap water for bathing with the option to use a mild cleanser that has a neutral pH (5.5 to 7.0). The absence and inconsistencies in guidelines are likely to be the result of a dearth of robust evidence from which to inform practice. [Correction added after online publication 19 Feb 2013: In the previous two paragraphs, the NICE references were erroneously listed as “NIHCE.” This has been corrected here and in the reference list.]

In a recent systematic review (Crozier & Macdonald, 2010) of newborn cleansing products versus water, of nine studies identified, only two were eligible for inclusion. A meta-analysis was not carried out because of the heterogeneity of trial protocols. The first study by Garcia Bartels et al. (2010) included 64 full-term newborns in Berlin and aimed to test the hypothesis that twice-weekly bathing with a commercially available wash gel and additional cream would not harm the natural adaptation of the skin barrier in healthy newborns. Participants were randomized to one of the four following trial arms: bathing twice weekly with commercially available wash gel product; bathing twice weekly with clear water, then applying a commercially available body cream; bathing with wash gel and applying cream after bathing; and bathing with clear water only. The second study included in the systematic review (Dizon, Galzote, Estanislao, Mathew, & Sarkar, 2010) was a three-armed trial conducted in the Philippines, which compared two different liquid cleansers with water alone; 60 infants were randomized in each arm. Authors of both studies concluded that neonatal skin barrier function was not harmed by the tested skin care regimens in healthy, full-term infants. However, neither provided an a priori primary outcome or sample size, nor did they follow the Consolidated Standards of Reporting Trials (CONSORT) guidelines (Begg et al., 1996) for reporting. Thus, the authors of the systematic review concluded that there is currently insufficient evidence on which to base practice.

The absence of adequately powered randomized trials led us to develop a research program to examine whether a bathing product formulated for infants is appropriate for newborn bathing. First, a qualitative study exploring the views of women and health professionals on bathing regimens, in one U.K. setting, was conducted (Lavender et al., 2009). This study confirmed the inconsistencies in bathing practices and the readiness of women to use bathing products. We then conducted a pilot randomized, controlled trial (Lavender et al., 2011) comparing a newborn bathing product with water alone. The aim of the pilot study was to inform decisions for the main trial design and to optimize the robustness of trial processes. The pilot study confirmed that the primary outcome measure (transepidermal water loss [TEWL]) was feasible. However, no trends in the data were found in any direction or on any site of the body. Therefore, we have proceeded with a noninferiority trial to test the hypothesis that the use of a wash product formulated for newborn (<1 month of age) bathing is not inferior (no worse) to bathing with water only.

## Methods

Ethical approval was obtained from the Cheshire Research Ethics Committee. All participants gave written and verbal consent to participate. Between February 2010 and March 2011, we recruited healthy newborn infants born at 37 weeks gestation or more from a teaching hospital in the Northwest of England. We excluded infants if they were admitted to the neonatal unit; were receiving phototherapy; had limb defects, nontraumatic impairment of epidermal integrity, or evidence of skin disorder at first visit. We also excluded infants with a chromosomal abnormality or other syndromic diagnosis and infants going for adoption.

Pregnant women of potentially eligible infants were supplied study information in the antenatal period via community and hospital clinics. In the postnatal period, the attending clinical midwives sought verbal permission for a research midwife to approach women who had been given prior information. The research midwife collected baseline details from all women supplying verbal and written consent, using a self-administered questionnaire. Family history of atopic eczema also was established at this stage (defined as at least one of father, mother, or sibling who has had a medical diagnosis of atopic eczema and who has had topical steroid treatment). Interpreters were available for non-English speaking women.

### Randomization and Follow-Up

Infants were randomized to a bathing regimen using a newborn wash product or water alone before their first bath. Randomization was by consecutively numbered, sealed, opaque envelopes held by the research manager at the study hospital. The randomization sequence was in uneven blocks and stratified according to whether there was a family history of atopic eczema. All women were given written instructions on how to bathe their infants. Parents were requested not to use any additional products, such as oils, sponges, and wipes. Participating mothers were instructed to bathe their neonate at least 3 times per week and to avoid rubbing of the skin. Those allocated to bathe their neonate in water alone (control) were not provided with any products. If these mothers wished to use shampoo on their neonates’ hair, they were requested to do this outside of the bath, and to ensure that the neonate's body was wrapped in a towel, to prevent contact with the skin. Those allocated to the wash product (experimental) were provided with sufficient newborn wash and advised to dilute the product at a ratio of three squirts per bath. On the day of assessment, mothers were requested to delay bathing their neonate until measurements had been taken.

Mothers have expressed concern that water alone is an ineffective cleanser, but evidence on appropriate newborn bathing has been insufficient to inform practice.

The chosen commercially available wash product, Johnson's Baby Top-to-Toe Bath (Johnson & Johnson Limited, Maidenhead SL6 3UG, UK), is a soap-free liquid cleanser designed for newborns’ skin. It is sodium lauryl sulphate-free and consists of a proprietary blend of nonionic and amphoteric surfactants that when combined result in large micelles that clean via dispersal of fats without disrupting the skin barrier. The formula contains well-tolerated preservatives and a low level of fragrance; it is pH-adjusted (around 5.5) and hypoallergenic. The International Nomenclature Cosmetic Ingredients list comprised aqua, coco-glucoside, cocamidopropyl betaine, citric acid, Acrylates/C10–30 Alkyl Acrylate Crosspolymer, sodium chloride, glyceryl oleate, p-Anisic acid, sodium hydroxide, phenoxyethanol, sodium benzoate, and parfum.

### Outcome Measures

We planned to examine objective markers of skin integrity that are sensitive enough to detect subclinical changes in skin barrier function. Our pilot trial established that obtaining baseline measurements in hospital and follow-up outcome measurements at home was feasible and acceptable to mothers (Lavender et al., 2011). On the basis of our pilot trial and previous work in this field, the primary outcome was based on measurements of TEWL, which is a measure of the flux of water vapor evaporating from the skin surface. This technique provides an overall marker of how an intervention or development affects the function of the skin as a barrier to water loss (Flohr et al., 2010); lower values represent a good skin barrier. This technique is noninvasive and has been measured in preterm and term neonates (Agren, Sjörs, & Sedin, 2006; Harpin & Rutter, 1983) and older infants (Nikolovski et al., 2008). The primary outcome measure was the average of TEWL measurements, using a closed chamber system, over three sites (outer forearm, midpoint between wrist and elbow; front of thigh, midpoint between knee and groin; abdomen, midpoint between umbilicus and sternum) at 14 days following birth using an AquaFlux Model AF200 (Biox Systems Ltd, London, UK). The pilot study (Lavender et al., 2011) found no important differences in TEWL measurements between body parts; therefore, we analyzed an average assessment score.

Secondary outcomes comprised TEWL at 4 weeks postbirth, skin surface pH using a Courage + Khazaka Skin-pH-Meter® PH 900, and stratum corneum hydration scores using Corneometer® CM 820 (Courage + Khazaka electronic GmbH, Cologne, Germany) from baseline (within 48 hours of birth). Because of the sensitivity of the neonate's skin in the early weeks following birth, this is an ideal time to investigate the effect of wash products. Any differences in these outcomes are likely to be greater than later in an infant's life when the skin barrier is more stable.

These secondary outcomes were measured at 2 and 4 weeks postbirth. Anatomical markers were used to ensure assessments were consistent. Two measurements were taken at each site; mean scores were used in analysis. Skin measurement instruments were calibrated before recruitment, during the recruitment period, and following the last follow-up assessment.

Clinical measures comprised observations of the skin daily by the mother from first assessment to 4 weeks postbirth; a semistructured diary was used to record observations. Observations were made by the midwife at the times of assessment and were recorded with the aid of a validated skin assessment scale (Lund & Osborne, 2004) that included a scale for erythema, excoriation, and dryness. For each component an average grade was obtained across body parts. This scale has been used in a number of comparative studies (Brandon, Coe, Hudson-Barr, Oliver, & Landerman, 2010; Garcia Bartels et al., 2011). Mothers’ satisfaction with their allocated cleansing regimen was obtained using a specifically designed questionnaire that had been evaluated for content validity through earlier qualitative work (Lavender et al., 2009).

### Blinding

The research midwives carrying out the measurements were blinded to treatment allocation. Women were asked not to reveal their allocation to the midwife assessing the neonate in their home. The research assistant telephoned women on the day of assessment to remind them to remove visible signs of products prior to the midwife's arrival.

### Compliance

To optimize compliance levels, weekly telephone contact with participating women was maintained. Participants were requested to keep a cleansing diary for the study duration. It was prespecified that we would assess the impact of any noncompliance on the primary outcome using five categories. *Strict compliance* was defined as nonuse of any additional products on the areas of the body under assessment or any products likely to cause contamination (e.g., talcum powder). *Mostly compliant* and *partially compliant* were defined as the nonuse of additional products in the week before the assessment, with no more than two product deviations and more than two product deviations, respectively. *Rarely compliant* and *noncompliant* were defined as use of additional products on the areas of the body being measured or any products likely to cause contamination during the week of assessment with no more than two product deviations and more than two product deviations, respectively.

### Statistical Analysis

Data were analyzed using Stata 11 (StataCorp LP, College Station, TX) following double entry of all data, with the statistician blinded to the true treatment allocation. Two data sets were defined that were used for analysis. The intention-to-treat (ITT) data set includes all the participants who have data for the primary outcome. The per protocol (PP) data set excludes participants satisfying ITT who were classified as rarely compliant or noncompliant.

Preliminary analysis compared the demographic and baseline characteristics of participants between treatment groups, further stratifying by family history of atopic eczema. A prespecified linear model was used to estimate the effect of treatment on the primary and secondary outcomes, adjusting for family history of eczema, baseline outcome, and state of neonate (awake and calm, asleep, crying) at baseline and follow-up. Preliminary blinded analysis suggested that the neonatal state may influence the outcome measurements; in particular, crying, which may be indicative of stress, is associated with movement and can affect skin surface temperature (Rogiers & EEMCO, 2001). Clinical outcomes were summarized by percentages and compared using odds ratios adjusted for randomization strata and associated *p* values. Maternal views of the intervention were compared between groups using Fisher's exact test or *t* test.

Multiple sensitivity analyses were carried out to test the effect of data and analysis assumptions and where any imbalance in demographic or baseline data was found.

One extreme outlying TEWL value (255) was assumed to be an instrument error and was treated as missing. Four neonatal states were missing at the 2-week follow-up and were assumed to be awake and calm. Sensitivity analysis investigated to what extent these assumptions affected the results. All significance levels are presented without adjustment for multiple testing.

### Sample Size

Based on the pilot data, we estimated that a non important inferiority in TEWL would be 1.2. The upper limit of subclinical difference was determined by experienced clinicians on the trial management group and Data Monitoring Committee. Assuming a common standard deviation is 3 and allowing for a very small actual difference of up to 0.2 units, favoring water, a sample size of between 100 and 140 per group would have 80% power to reject the null hypothesis that the test and standard are not equivalent (the average TEWL value for product is at least 1.2 units greater than that for water), based on a two-group, 2.5%, one-sided *t* test. Assuming a 10% dropout over 4 weeks, and an actual difference of 0.1 units we aimed for 120 completers per group by recruiting at least 133 participants per group at baseline. Because the loss to follow-up was greater than anticipated, we over-recruited to compensate.

## Results

**Figure 1:**
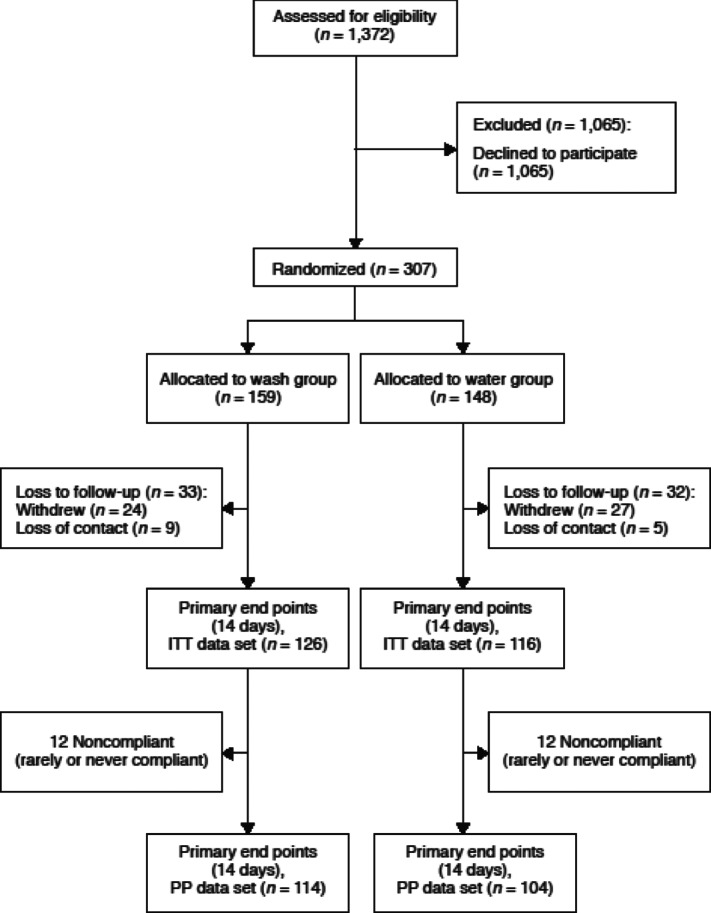
Participant flow during the study.

Overall, 307 infants were randomized and the participation rate was 22.4% (307/1372). The overall loss to first follow-up was 65/307 (21.2%); 33 (10.7%) in the wash group and 32 (10.4%) in the water group. Participant flow can be seen in Figure 1. By first follow-up, 109/159 participants in the wash group were strictly compliant with the study protocol, 10 were mainly compliant, 3 were partially compliant, 9 were rarely compliant, and 4 were noncompliant; one participant complied with the wrong treatment allocation. In the water group, 92/148 participants were strictly compliant, 11 were mainly compliant, 5 were partially compliant, 9 were rarely compliant, and 3 were noncompliant; one participant complied with the wrong treatment allocation. Similar baseline characteristics (Table 1) and all first skin assessment measures also were comparable between randomized groups (Table 2).

**Table 1:** Baseline Characteristics of Participants by Randomized Group

Characteristic	Wash	Water
	(*n* = 159)	(*n* = 148)
Family history of atopic eczema	42 (27)	42 (29)
Maternal age (years), mean (*SD*)	28.6 (5.6)	28.2 (6.1)
Maternal ethnicity		
White, British	128 (81)	132 (89)
Black and ethnic minority	16 (10)	14 (10)
Other	15 (9)	2 (1)
Maternal parity		
Primiparous	79 (50)	76 (51)
Multiparous	80 (50)	72 (49)
Birth mode		
Normal vaginal	115 (72)	99 (67)
Instrumental	20 (13)	25 (17)
Caesarean section	22 (14)	24 (16)
Vaginal breech	2 (1)	0 (0)
Infant's gender		
Male	80 (50)	78 (53)
Female	79 (50)	70 (47)
Birth weight, g	3435 (429)	3445 (525)
Feeding method		
Breast	65 (41)	53 (36)
Bottle	76 (48)	80 (54)
Mixed	18 (11)	15 (10)

*Note*. Values are *n* (%) unless stated otherwise.

**Table 2:** First Skin Assessments by Randomized Group

Measurement, mean (*SD*)	Wash	Water
	(*n* = 159)	(*n* = 148)
TEWL (g/m^2^/h)	13.1 (3.4)	13.3 (3.6)
Hydration (arbitrary units)	37.6 (9.4)	36.1 (10)
Skin pH	6.2 (0.6)	6.2 (0.6)

Note. TEWL = transepidermal water loss.

### Primary Analysis

**Figure 2:**
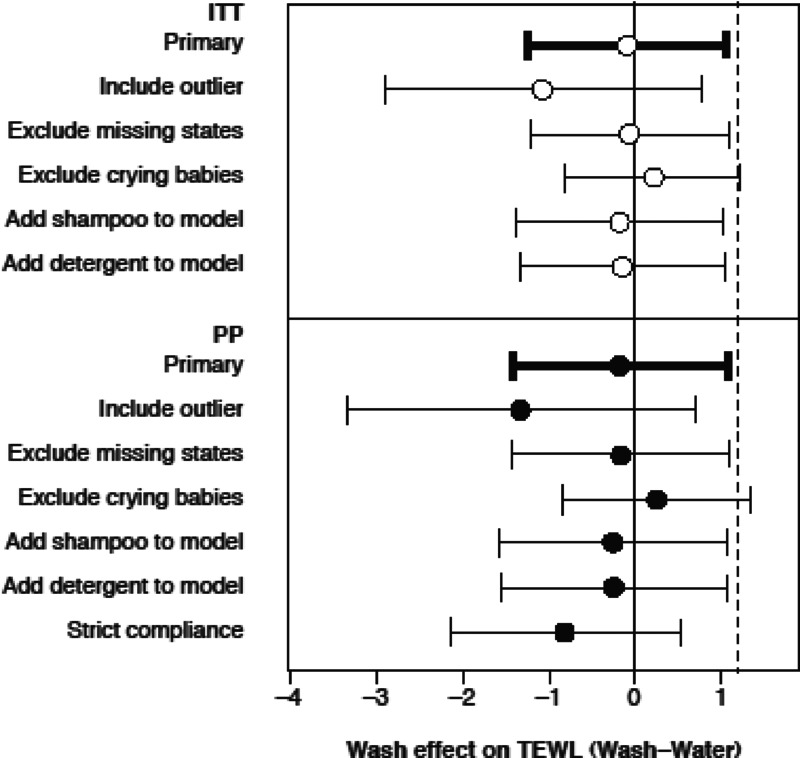
Sensitivity analyses: 95% confidence interval for difference in TEWL score at 2 weeks between groups for a number of different alternative assumptions and analyses. ITT = intention-to treat; PP = per-protocol; TEWL = transepidermal water loss.

There was very little difference between the wash and water groups in terms of TEWL (Table 3), and the upper limit of the 95% confidence interval of the difference did not reach the prespecified noninferiority margin. The location of the 95% confidence interval (CI) in relation to the prestated noninferiority value shows that the study was adequately powered. Following the primary analysis, sensitivity analysis was carried out including the one outlying TEWL value (deemed to be an instrument error); excluding the four with missing neonatal state (assumed to be awake and calm); excluding all crying infants, further adjusting for shampoo and detergent use at first follow-up; and for the PP analysis only including strictly compliant (the five level compliance definition) (Figure 2). None of these analyses substantially altered the results.

**Table 3:** Comparison of Primary Outcome Measure (TEWL) at Follow-Up by Randomized Group Under ITT and PP Analyses

Analysis	Wash (*n* = 126)	Water (*n* = 116)	Wash–water adjusted difference	*p*-value
	Mean (*SD*)	Mean (*SD*)	in means (95% CI)^a^	
ITT	12.8 (4.5)	12.5 (5.6)	−0.08 (−1.24, 1.07)^a^	.89
PP	12.8 (4.6)	12.6 (5.9)	−0.17 (−1.42, 1.09)^a^	.79

*Note*. CI = confidence interval; ITT = intention-to-treat; PP = per protocol; TEWL = transepidermal water loss.

a Controlling for family history of eczema, infant state at 2 weeks and baseline, and baseline TEWL.

### Secondary Analyses

Tables 4, 5, and 6 present the secondary outcomes that detail the biophysical assessment, clinical observations, and maternal views.

**Table 4:** Comparison of Secondary Biophysical Outcomes at Follow-Up by Randomized Group (ITT Analyses)

Analysis, mean (SD)	Wash	Water	Wash–water adjusted difference	*p* value
	(*n* = 126)	(*n* = 116)	in means (95% CI)^a^	
14 Days				
pH	5.5 (0.5)	5.5 (0.5)	0.05 (−0.07, 0.17)	.40
Hydration	49.7 (9.2)	46.7 (8.4)	2.9 (0.43, 5.47)	.022
28 Days				
TEWL	12.6 (5.2)	13.6 (5.4)	−0.90 (−2.20, 0.40)	.17
pH	5.8 (0.5)	5.3 (0.4)	0.03 (−0.09, 0.16)	.60
Hydration	54.1 (9.9)	54.7 (9.3)	2.4 (−0.40, 5.20)	.093

*Note*. CI = confidence interval; ITT = intention-to-treat; TEWL = transepidermal water loss.

a Controlling for family history of eczema, infant state at 2 weeks and baseline, and baseline TEWL.

**Table 5:** Comparison of Clinical Observations by Randomized Group (ITT Analyses)

		14 Days		OR (95% CI)^a^	*p*-value	28 Days		OR (95% CI)^a^	*p* value
							
		Product	Water			Product	Water		
		*n* = 126	*n* = 116			*n* = 126	*n* = 104		
		*n* (%)	*n* (%)			*n* (%)	*n* (%)		
Dryness	None	74 (58.73)	56 (48.28)	0.66^b^ (0.40, 1.09)	.11	102 (80.95)	93 (89.42)	1.74 (0.79, 3.82)	.17
	Dry	47 (37.30)	56 (48.28)			21 (16.67)	11 (10.58)		
	Very dry	4 (3.17)	2 (1.72)			0 (0.00)	0 (0.00)		
	Missing	1 (0.79)	2 (1.72)			3 (2.38)	0 (0.00)		
Erythema	None	116 (92.06)	98 (84.48)	0.46 (0.20, 1.05)	.07	111 (88.10)	92 (88.46)	0.83 (0.36, 1.93)	.66
	<50% body	9 (7.14)	16 (13.79)			12 (9.52)	11 (10.58)		
	surface								
	>50% body	0 (0.00)	0 (0.00)			0 (0.00)	1 (0.96)		
	surface								
	Missing	1 (0.79)	2 (1.72)			3 (2.38)	0 (0.00)		

*Note*. CI = confidence interval; ITT = intention-to-treat; OR = odds ratio.

a Wash relative to water.

b Dry and very dry skin data combined.

**Table 6:** Maternal Views of Bathing Regimen (ITT Analyses)

	Product (*n* = 111)	Water (*n* = 99)	Fisher's Exact
	*n* (%)	*n* (%)	*p* value
Smell			<0.001
Made infant smell nice	73 (65.77)	26 (26.26)	
Made no difference	38 (34.23)	68 (68.69)	
Made infant smell unpleasant	0 (0.00)	5 (5.05)	
Missing	0 (0.00)	0 (0.00)	
Cleanliness			0.59
Cleaned adequately	97 (87.39)	76 (76.77)	
Did not clean adequately	7 (6.31)	8 (8.08)	
Made no difference^a^	6 (5.41)	0 (0.00)	
Missing	1 (0.90)	15 (15.15)	
Dryness			0.66
Made skin more dry	8 (7.21)	10 (10.10)	
Made no difference	65 (58.56)	53 (53.54)	
Made skin less dry	38 (34.23)	36 (36.36)	
Missing	0 (0.00)	0 (0.00)	
Would participate in trial again			0.87
Yes	107 (96.40)	95 (95.96)	
No	1 (0.90)	2 (2.02)	
Unsure	3 (2.70)	2 (2.02)	
Missing	0 (0.00)	0 (0.00)	
Recommend method of bathing to a friend?			0.47
Yes	95 (85.59)	86 (86.87)	
No	6 (5.41)	8 (8.08)	
Unsure	10 (9.01)	5 (5.05)	
Missing	0 (0.00)	0 (0.00)	
Quality of trial Information			1.0
Good	101 (90.99)	91 (91.92)	
Adequate	10 (9.01)	8 (8.08)	
Poor	0 (0.00)	0 (0.00)	
Missing	0 (0.00)	0 (0.00)	
Still using the same regimen?			0.14
Yes	80 (72.07)	62 (62.63)	
No	30 (27.03)	37 (37.37)	
Missing	1 (0.90)	0 (0.00)	
If so, do you intend to continue regimen?			0.010
Yes	67 (60.36)	41 (41.41)	
No	7 (6.31)	16 (16.16)	
Data not required^b^	7 (6.31)	5 (5.05)	
Missing	30 (27.03)	37 (37.37)	
Satisfaction rating			0.15
10 cm visual-analogue scale	9.12 (1.52)	8.82 (1.56)	
Missing	2 (1.80%)	1 (1.01%)	

*Note*. ITT = intention-to-treat.

a This category was omitted from the statistical comparison as its meaning is ambiguous.

b Data was not required for the 67 participants who answered “no” to “same regimen.”

*Biophysical Assessments*. Skin assessment results are presented in Table 4. No significant differences were found in the pH measurements at 14 days, as well as no significant difference in TEWL at 28 days. Differences were found in stratum corneum hydration that was higher in the wash group at 14 days. However, after adjustment for multiple testing, this difference would not be considered statistically significant and was reduced at 28 days.

*Clinical Observations*. Clinical observation results are presented in Table 5. Observations of dry and very dry skin were merged for analysis because of the limited information that the very dry category held. No significant differences between groups were found when observing for dryness or erythema, and no excoriation was observed in either group.

*Maternal Views*. Mothers’ perception of their allocated skin regimen, gathered using diaries for the intervention period and questionnaires at 8 weeks, are presented in Table 6. Two-hundred-and-ten participants responded; 68.4% of the initial 307 in the trial. The response was representative of the original sample, with approximately 53% in the product arm and 47% in the water arm (compared with 52% and 48% in the original sample). Differences between groups were only found in two items assessed by mothers. Mothers were more likely to state that using a bathing product left their neonate smelling good (*p* < .001). This echos the findings of previous qualitative work, in which mothers indicated that smell was a factor in product choice (Lavender et al., 2009). Mothers in the wash group who continued their allocated regimen post 28 days were more likely to maintain the same bathing regimen following study completion (*p* = .010). There were no differences in overall satisfaction scores, as assessed by a 10-cm visual analog scale.

## Discussion

We reported findings from the largest randomized clinical trial of healthy newborn infants and bathing, demonstrating that the cleansing product tested in our trial is not inferior (no worse) to bathing with water alone, as assessed by TEWL. Maintenance of correct water balance is one of the key functions of the epidermis (Harpin & Rutter, 1983; Hoath & Narendran, 2001). Increased TEWL precedes the development of AD in those with a genetic predisposition to the development of AD and is therefore a clinically relevant measure (Flohr et al., 2010). Despite stratifying for a family history of atopic eczema, we found no difference in TEWL measurements between those bathed in water alone or with a cleansing product, offering reassurance that the cleansing product was not influencing skin barrier integrity. This study demonstrates that the product tested in this trial is acceptable and safe for use on newborn skin.

In the largest randomized trial of healthy newborns, no differences were found between using a specified proprietary wash product and using water alone.

In our earlier work (Lavender et al., 2011), mothers raised concerns regarding skin dryness; this was not raised as an issue in this trial. Although there are known changes in hydration as the infants’ skin matures (Nikolovski et al., 2008), this was a randomized, controlled trial; and these changes were expected to be similar between trial groups. Stratum corneum hydration was higher in the wash group at 14 days, which appears significant on an unadjusted basis; however, this difference would not be considered statistically significant after adjustment for multiplicity. Furthermore, any differences observed had disappeared by 28 days, suggesting that this may be an unimportant or even a chance finding. This is an area that warrants further study, especially as bathing the skin in water may elute out natural moisturizing factor (NMF) from the skin. Natural moisturizing factor has an important role as a humectant, retaining water within the stratum corneum. Natural moisturizing factor also contains several acids, including lactic acid, urocanic acid, and sodium pyrrolidone carboxylic acid. These acids keep the pH of the stratum corneum low, which is important for the maintenance of the normal skin barrier. In order to reduce the eluting of NMF from the stratum corneum, bathing should be restricted to the shortest period possible (e.g., 5 minutes).

Overall, maternal satisfaction between trial arms showed no differences. However, importantly, more than two thirds of women who were allocated to the wash group were still using their allocated regimen at 8 weeks postbirth. Of these, 60% intended to continue with this regimen, suggesting satisfaction with the product. Moreover, it confirms our earlier findings (Lavender et al., 2009) that many parents prefer to use bathing products versus water alone. These findings offer evidence to recommend that parents should be supported to use their preferred method of neonatal bathing.

We found no evidence of harm with the use of the product evaluated in this study. These findings resonate with those of other recent trials (Dizon et al., 2010; Garcia Bartels et al., 2010) that also failed to find evidence of harm when using modern cleansing products. Unlike the previous studies, this trial stated an a priori primary outcome, ensured the study was adequately powered, and was distinguished from previous work by quality and sample size. Our work is a significant advancement in the evaluation of skin care in the month after birth.

This study was conducted in one hospital setting. Nevertheless, this setting supports women from diverse sociocultural backgrounds and our data reflect this; findings are likely to be transferable to other settings. However, further research may be needed in settings where cultural practices, environmental conditions, or water conditions are different; all of which may influence TEWL measurements.

Parents are faced with a multitude of commercial products and have to navigate the complex ingredient information and influential branding before making purchasing choices. Although the choice of products can vary around the world, certain minimum criteria should be met when they are indicated for use in newborns and infants. These include not only appropriateness of pH, around 5.5, and documented skin safety, but also more importantly, ocular safety because cleansers can often be inadvertently splashed into a neonate's developing eyes during routine use. Increasingly, parents are seeking the evidence to support their child care practices, evidenced by the increasing numbers of websites and parental “hits.” The term *clinically tested*, which appears on many neonatal products, covers a range of testing and fails to provide accurate detail on the rigor of such investigations. New regulations are needed to cover the claims that can be made for a particular product in relation to the quality of the clinical trial evidence. This would allow parents to identify the best products and encourage companies to invest in producing the best formulations and evaluating them in appropriately powered clinical trials. In our opinion, to ensure that truly informed decisions are made, parents should be informed of neonatal products that have been tested in robust randomized clinical trials. Nurses and midwives have a key role in providing such information during parent education sessions. Nurses have a professional responsibility to critique new evidence, which sometimes supersedes national guidelines, particularly when the guidelines have been based on low levels of evidence.

The findings of this trial will enable health professionals to provide women with evidence on which to base their choice for newborn cleansing.
